# Acknowledgment to the Reviewers of *International Journal of Neonatal Screening* in 2022

**DOI:** 10.3390/ijns9010005

**Published:** 2023-01-18

**Authors:** International Journal of Neonatal Screening Editorial Office

**Affiliations:** MDPI AG, St. Alban-Anlage 66, 4052 Basel, Switzerland

High-quality academic publishing is built on rigorous peer review. *International Journal of Neonatal Screening* was able to uphold its high standards for published papers due to the outstanding efforts of our reviewers. Thanks to the efforts of our reviewers in 2022, the median time to first decision was 21 days and the median time to publication was 49 days. Regardless of whether the articles they examined were ultimately published, the editors would like to express their appreciation and thank the following reviewers for the time and dedication that they have shown *International Journal of Neonatal Screening*:
Aishwarya, SiddharthMarcão, AnaAlcántara-Ortigoza, Miguel AngelMessina, Maria AnnaAnderson, SharonMillington, David S.Argudo Ramírez, AnaMincarone, PierpaoloArmstrong, Kate L.Moat, StuartAsamoah, AlexanderMonostori, PéterBakkeheim, EgilMunck, AnneBasheeruddin, KhajaNakamura-Utsunomiya, AkariBhattacharjee, ArindamOrsini, JoeBonham, JimPalasanthiran, PamelaBorrajo, Gustavo J. C.Palma, SilviaBrockow, InkenParad, Richard B.Bush, Lynn WeinPesch, MeganCabello, Juan FranciscoPitt, JamesCarling, RachelPng, May EeCocho, José A.Purcell, Patricia L.Cordovado, Suzanne KehoeRegelmann, Molly O.Coughlin, Curtis R.Ribes, AntoniaCunningham, AmyRizzo, CristianoDangouloff, TamaraRizzo, WilliamDe Hora, Mark R.Rocha, HugoDe Winter-de Groot, Karin M.Rock, Michael J.Dekkers, Eugenie H. B. M.Ruoppolo, MargheritaDhiman, VineetScharfe, CurtDluholucký, SvetozárSchulze, AndreasEyskens, FrancoisSciortino, StanleyFarrell, Philip M.Servais, LaurentFicicioglu, CanShone, ScottFingerhut, RalphSicko, Robert J.Franková, VěraSomech, RazGaviglio, AmyStepien, Karolina M.Gibson, James B.Tajima, GoGoldenberg, AaronTang, HaoGrosse, Scott D.Tangeraas, TrineHall, KateTavakoli, Norma P.Hall, PatriciaTaylor, JenniferHeather, NatashaTerlizzi, VitoHinton, Cynthia F.Tewari, Vishal VishnuHwu, Wuh-LiangTherrell, BradfordIshige, NobuyukiTluczek, AudreyJalali, AliVan Vliet, GuyJones, KendraVeraguth, DorotheKemper, Alex R.Vockley, Cate WalshKimizu, TomokazuVon Döbeln, UlrikaKingsmore, Stephen F.Wang, RaymondKucera, Katerina S.Webster, DianneLilian, DownieWedell, AnnaLund, Allan MeldgaardWiberley-Bradford, Amy E.Mak, Chloe MiuWiley, VeronicaMallack, EricYahyaoui, RaquelManning, AdrienneZetterström, Rolf


